# What barriers and facilitators to self-management are experienced by mothers who wish to breastfeed but require concurrent pharmacotherapy in the first two years postpartum? A mixed-methods systematic review protocol.

**DOI:** 10.12688/hrbopenres.14100.2

**Published:** 2025-11-21

**Authors:** Lucy Hackett, Deirdre M. D'Arcy, Juliette O'Connell, Samuel Cromie, Déirdre Daly, Tamasine Grimes

**Affiliations:** 1 School of Pharmacy and Pharmaceutical Sciences, Panoz Institute, Trinity College Dublin, The University of Dublin, Dublin 2, D02 PN40, Ireland; 2 School of Psychology, Áras an Phiarsaigh, Trinity College Dublin, the University of Dublin, Dublin 2, Ireland; 3 School of Nursing and Midwifery, Trinity College Dublin, The University of Dublin, 24 D’Olier Street, Dublin 2, D02 T283, Ireland; 4 Trinity Centre for Maternity Care Research (TCMCR), School of Nursing and Midwifery, Trinity College Dublin, The University of Dublin, 24 D’Olier Street, Dublin 2, D02 T283, Ireland

**Keywords:** Medication, pharmacotherapy, breastfeeding, infant feeding, postpartum, experience

## Abstract

**Background:**

Breastfeeding is the recommended method of infant feeding. The postpartum period can be a challenging time for women who experience illness requiring pharmacotherapy. However, breastfeeding women may use medication less frequently than their counterparts who are not breastfeeding. Some women report the need for pharmacotherapy as a reason for earlier than desired breastfeeding cessation. The experiences of women in relation to self-management of medication use and breastfeeding are poorly understood.

**Aim:**

The aim of this systematic review is to explore the barriers and facilitators to self-management for women who wish to breastfeed but require concurrent pharmacotherapy in the first two years postpartum, using mixed-methods and a systems-based theoretical framework.

**Methods:**

Systematic searches will be performed using six electronic bibliographic databases. Dual independent selection, data extraction and quality assessment of studies will be carried out. A convergent integrated approach to data synthesis will be used. The System Engineering Initiative for Patient Safety (SEIPS) model will be used as a theoretical framework to guide data synthesis. Input and collaboration from public and patient involvement (PPI) contributors will be sought throughout.

**Conclusion:**

Enhancing understanding of mothers’ self-management experiences when they wish to breastfeed and take medication is arguably key to improving maternal and child health and quality of life. The proposed review will synthesise the available data regarding the barriers and facilitators to self-management for women who face the need for concurrent pharmacotherapy and breastfeeding. In doing so, important supports and unmet needs of this cohort will be revealed.

**PROSPERO registration number:**

CRD420251000918. Amendments to this protocol will be uploaded as revision notes to any platforms where the protocol was published.

## Background

Breastfeeding is recommended due to the benefits it offers both babies and their mothers. Globally, 48% of infants under 6 months of age are exclusively breastfed ^1^. Breastfeeding rates tend to be lower in high-income countries compared to low- and middle-income countries ^2^. In Ireland, for example, breastfeeding rates are among the lowest in the world ^3^. Despite limited data, it is reported that only 15% of babies are exclusively breastfed to 6 months of life in Ireland ^4^. The reasons behind low breastfeeding rates are often multi-faceted, however some women report the need to take medication as a reason for earlier than desired breastfeeding cessation ^5– 7^.

Most women will require some form of medication during the first two years after giving birth. The postpartum period may be a challenging time for women, with many reporting that they experience pain, infection, frequent minor illnesses, severe headaches or migraines, and persistent mental health problems ^8, 9^. Other women may have acute or chronic health conditions. Pharmacotherapy is often the first-line treatment for many of these issues. Studies report that between 49–96% of women use some form of medication during breastfeeding ^10– 13^, however breastfeeding mothers are reported to use medication less frequently than their counterparts who are not breastfeeding ^10^.

Medication use for the management of chronic conditions has been shown to decline in pregnancy and remains low in the months following, most likely due to breastfeeding ^14^. This may suggest that some women will prioritise infant feeding over their own health if they perceive that these goals are incompatible. It has been reported that women who discontinue pre-existing medications while breastfeeding have shorter breastfeeding duration and are less likely to meet their personal breastfeeding goals than women who breastfeed while continuing effective medications ^15^. This is a highly unfavourable situation, as women may suffer morbidity due to medication discontinuation, while also not benefiting from the protective effects of continued breastfeeding for themselves or their infants.

Women may be fearful to take medication while breastfeeding due to concerns about medication transfer to the infant through the breastmilk ^16, 17^. This may result in them refraining from either breastfeeding or taking required medication, or doing one of these actions in an impractical manner ^18, 19^. While a small number of medications are accepted as contraindicated during breastfeeding ^20– 22^, many medications needed during this period may be considered safe or to have a safe alternative; there are a relatively small number of adverse events reported in breastfed infants whose mothers have taken medication ^23^ and the amount of drug transferred to the infant via the breastmilk is usually too small to exert any effect ^24^. However, deciphering which medicines are safe may be a difficult task.

Fear regarding decision-making in this setting may stem from the fact that there is less safety data available regarding pharmacotherapy for pregnant and breastfeeding women compared to the general population. These cohorts are usually excluded from drug trials and research, with current evidence often based on case studies that have evolved over time ^25^. Therefore, despite the likely safety of (or safe alternatives for) many medications ^23, 24, 26^, few drugs are licenced for use in lactation and thus it may be perceived that either breastfeeding or pharmacotherapy cannot go ahead due to the risk to the infant ^23, 27^. However, decisions regarding the relative safety of drugs in lactation may be made in many cases by considering factors such as infant age, the frequency of breastfeeding, concurrent infant disease, whether the medication is licenced for paediatric use, and pharmacokinetics ^22, 26, 27^. Thus, advice and decision-making regarding pharmacotherapy during lactation is complex and needs to be individualised. To do this however, prescribers may need to seek out specialised literature and take professional responsibility for their clinical judgement in these situations ^22, 27^.

Furthermore, there is a potential for some medications to have physiological effects on lactation and infant behaviour. This has been noted in the literature in relation to antidepressants ^28^ and intrapartum synthetic oxytocin ^29– 31^, for example. Little is known about whether and how this may impact medication self-management and women’s choices when pharmacotherapy and breastfeeding coincide. The effects of medication on lactational physiology and infant behaviour may be one consideration among many for these women.

Medication self-management is the series of steps a person takes to use their medication safely and effectively ^32^. These steps may fall under the definition of “patient work” ^33, p1676^. Breastfeeding women may encounter difficulties in relation to medication self-management due to their own, or healthcare professionals’ (HCPs), concerns regarding the transfer of medicines through breastmilk.

Existing literature reviews ^18, 34^ have sought to investigate the knowledge, attitudes, practices and behaviours of women and HCPs regarding medication use during breastfeeding. While the literature outlines the perceived “safety behaviours” ^18, p111^ that women may adopt, these behaviours cannot be understood due to the lack of literature detailing women’s knowledge and attitudes regarding medication use while breastfeeding ^18^. The proposed review will help to address this gap in the literature by providing insight into the barriers and enablers to effective and concurrent pharmacotherapy and breastfeeding as experienced by women. Neither of these literature reviews have applied a systematic review methodology, which further emphasises the need for the proposed study. A recently published systematic review however ^35^, did explore one possible outcome for women who face concurrent breastfeeding and medication needs. This review aimed to identify the percentage of women who discontinue breastfeeding due to pharmacotherapy, which medications may trigger this, and other influencing factors. Authors of this review highlight the need for future research to explore women’s perspectives and experiences in these situations, their contact with healthcare professionals and services, and the wider social or cultural contexts which may influence outcomes. Pilgrim and colleagues also suggest that future research use mixed methods designs and a theoretical framework to understand the determinants of women’s decision-making when faced with the need for medication while breastfeeding. The proposed review seeks to address this gap in the literature, and to address all possible outcomes and self-management decisions for women who face the simultaneous need for breastfeeding and medication, including avoidance or discontinuation of medication, or methods aiming to reduce infant exposure to medication through breastmilk.

While previous reviews ^18, 34^ have sought to examine features of the individual (e.g. knowledge, attitudes, behaviours), the proposed review will use the Systems Engineering Initiative for Patient Safety (SEIPS) model ^33, 36, 37^ to examine the experiences of women within the context of the system around them. The complexity of self-management for women who wish to breastfeed and require pharmacotherapy may be best understood in this context. Such understanding may better explain women’s knowledge and behaviours in this setting and reveal potential research and innovation opportunities to enhance breastfeeding, medication safety and maternal experience.

### Rationale for the research

Supporting safe and effective breastfeeding and medication use may have significant returns for maternal, child and global health. To make this a reality, the barriers and facilitators to effective and concurrent pharmacotherapy and breastfeeding, as experienced by women, must be understood. Through the lens of the SEIPS model, opportunities for support and innovation may be revealed, which will facilitate medication optimisation through improved medication safety and maternal medication self-management. It may also enhance maternal experience, as well as rates of breastfeeding initiation and duration. A systematic review addressing this topic, designed in collaboration with women themselves, is overdue. 

A search of PROSPERO and Medline did not identify any previous systematic review on this topic.

## Aims and objectives

The aim of this systematic review is to explore the barriers and facilitators to self-management for women who wish to breastfeed but require concurrent pharmacotherapy in the first two years postpartum, using mixed-methods and a systems-based theoretical framework. The objectives are to:

1. Comprehensively synthesise the available data from quantitative, qualitative and mixed-methods studies relating to self-management for women who wish to breastfeed and take medication.
2. Use the SEIPS framework to identify the component parts of maternal self-management of breastfeeding and medication use, and map the interactions between them. Use this to develop an understanding of the barriers and facilitators to safe and effective medication use while breastfeeding, and the barriers and facilitators to breastfeeding while undergoing pharmacotherapy, as experienced by mothers.
3. Identify potential research and innovation opportunities across the system to improve women’s experiences, medication safety, breastfeeding rates, and maternal and child health.

## Methods

This protocol was developed in line with the Preferred Reporting Items for Systematic Reviews and Meta Analyses checklist for protocols (PRISMA-P) ^38, 39^.

 A mixed-methods systematic review has been chosen as most appropriate to investigate this research question to ensure that all relevant literature is integrated, allowing for a comprehensive understanding of the complexities of this topic. Collating evidence from quantitative, qualitative, and other mixed-methods research studies may allow for different aspects and perspectives of the same issue to triangulated in one review ^40^. This approach may also facilitate the findings of one type of data to be explained or contextualised by the other type of data ^40^.

The PEO (Population, Exposure, Outcome) tool is used to frame the research question ^41^.

*Population:* Studies which focus on the perspectives of mothers who breastfed their child(ren) at any point in the first two years of life, or women who considered breastfeeding. Studies which exclusively focus on the perspectives of healthcare professionals or other individuals will be excluded.
*Exposure*: Studies which address the need for simultaneous breastfeeding and pharmacotherapy. This may be understood as breastfeeding women who considered taking medication, or women who were undergoing pharmacotherapy and then considered breastfeeding. Medication in this context may be prescription-only medication, over-the-counter medication, or dietary supplements.
*Outcome:* The barriers and facilitators (or related concepts) to safe and effective self-management of medication use and breastfeeding, as experienced by mothers who face the need for these actions to be done concurrently.

### Search strategy

Six electronic databases will be systematically searched: CINAHL (Cumulative Index to Nursing and Allied Health Literature), EMBASE (Excerpta Medica Database), PsycINFO, Medline (Ovid), Maternity and Infant Care, and Global Index Medicus. Databases will be searched using terms related to medication, breastfeeding, women, experience, barriers, and facilitators. Searches will be limited to peer reviewed, empirical studies published in the English language, and not limited by year. Other literature reviews will be excluded. The search strategies used for all six databases are available ^39^. Additionally, literature will be hand-searched from identified pertinent systematic reviews or included studies by checking for relevant cited or citing papers.

### Screening and selection

Retrieved results will be exported to EndNote software and duplicates removed. The remaining results will then be exported to Covidence, a management software for systematic reviews for further de-duplication, followed by screening and selection for inclusion in the review.

Screening and selection of studies will be conducted in two rounds and presented using a Preferred Reporting Items for Systematic Reviews and Meta-Analysis (PRISMA 2020) flowchart in the review ^42^.

Round 1 will comprise title and abstract screening. Dual independent screening will be carried out by two members of the research team to reduce bias and ensure high quality screening. Disagreements will be resolved by consensus between the two reviewers or following input from a third reviewer if required.

Round 2 will comprise full text screening. Again, results will undergo dual independent screening by two researchers. An additional reviewer may be added if necessary to facilitate screening in a timely manner. Any discordance regarding the inclusion of a paper at this stage will be resolved by consensus between the two reviewers or following input from a third reviewer from the research team if required.

### Data extraction

Data from relevant studies will be extracted and evaluated to answer the review question using a standardised data extraction form which was produced using the PEO and SEIPS frameworks ^39^. In this form, details about the study characteristics, the participants, the context of medication use while breastfeeding, and the barriers and facilitators to self-management will be grouped under the headings of the PEO and SEIPS frameworks. This form will be piloted on five studies identified from the list of included studies in the review and refined following this if necessary. This will be formatted using Covidence software and will allow for dual data extraction. Authors of included papers will be contacted if necessary for clarification purposes or to request missing data. Data will be extracted primarily by one researcher (LH), and a sample of this cross-checked by a second member of the research team. Any disagreements between the two researchers will be resolved through discussion and, if necessary, the inclusion of a third member of the research team.

### Quality assessment

The Mixed-Methods Appraisal Tool (MMAT) ^43^ will be used to guide the assessment of included studies for methodological quality. This will be undertaken by one researcher (LH), with dual independent appraisal by another member of the research team. The quality rating of each study will be assessed by assigning a methodological response rating of ‘yes’, ‘no’, or ‘can’t tell’ to the quality criteria. Quality assessment results will be compared between the two researchers, and discrepancies resolved through discussion or with the involvement of a third researcher from the team. Studies will not be excluded for low methodological quality, but the methodological quality will be reported and considered in the findings and discussion sections of the review. Similarly, limitations or potential biases of included studies will be acknowledged.

### Data synthesis

The Joanna Briggs Institute methodological guidance for mixed-methods systematic reviews ^40^ will be followed for data synthesis in this review. The proposed review question may be answered by both qualitative and quantitative research; therefore, data synthesis will follow a convergent integrated approach. Dependent on the results of the systematic search, the most suitable approach for data transformation will be chosen. This data will then be combined to allow for further analysis.

The SEIPS model will be used as a theoretical framework to guide data synthesis ^33, 36, 37^. Deriving from the field of human factors and ergonomics, this framework seeks to illustrate how work systems influence health-related outcomes ^36^. The proposed review will use the SEIPS model to investigate the work systems, processes and outcomes relating to medication use while breastfeeding, how the components of these interact with one another and how this is experienced by women. 

The SEIPS 2.0 model ^33^ will primarily be used as it has a distinct focus on patient engagement and patient work, which is highly relevant to the concept of maternal self-management of medication use and breastfeeding. Some concepts may also be drawn from SEIPS 3.0 ^37^, which has a unique focus on the patient journey and experience, as well as care coordination within the healthcare system. The SEIPS framework will guide deductive analysis in the proposed review. Should any data fall outside of this model, inductive analysis may be required. If data from the literature permits, analysis will differentiate between occasional and chronic medication needs, as well as polypharmacy and single medication use, and how each of these are associated with breastfeeding. Other subgroups, such as infant age categories, will also be considered if possible.

### Public and Patient Involvement

Public and patient involvement (PPI) will be valued throughout the development of the systematic review in line with the Authors and Consumers Together Impacting on eVidencE (ACTIVE) framework ^44^. Past participants of the Maternal health and Maternal Morbidity in Ireland (MAMMI) study who had agreed to be contacted for future projects were approached regarding this study, and a PPI panel formed. Members of this panel were consulted prior to publication of this protocol for their input and validation of the research question. They will be offered opportunities to be involved in different stages of the research process such as data interpretation or the dissemination and outreach activities related to the study. Collaboration between the PPI panel and the research team will be key to analysing the barriers and facilitators experienced by women and co-developing a list of potential research and innovation opportunities to enhance medication safety during breastfeeding. The feedback and views of the PPI panel will be invaluable for the production and dissemination of a high-quality and impactful systematic review which focuses on a relevant research question.

### Dissemination plan

This mixed-methods systematic review will be submitted for publication to a relevant high-impact, top-percentile journal. Findings will be shared at relevant research conferences and promoted using internet and social media platforms. A strategy will be designed for sharing the findings with the public. Outreach activities may, for example, include meetings with relevant maternal advocacy or breastfeeding groups, or a free online webinar for women. Lay summaries and infographics will be developed for these activities, and consideration will be given to optimising accessibility for people with all levels of health literacy. Dissemination material will be co-designed and co-presented with PPI contributors to ensure that information and resources are accessible and engaging for the women that this research will affect. Dissemination will be considered throughout the lifetime of the project.

## Conclusion

Effective and appropriate medication use can allow women to lead healthier and better-quality lives. Similarly, encouraging and supporting more women to initiate and continue breastfeeding will have significant returns for maternal, child and global health. Empowering and facilitating concurrent breastfeeding and appropriate medication use, where safe, may allow for optimisation of maternal and child health and wellbeing. Such a reality requires an in-depth understanding of what women currently perceive as barriers and facilitators to self-management of breastfeeding and medication use. The proposed systematic review will address this gap in the literature and seek to identify relevant research and innovation opportunities.

## Time frame and current review status

The review protocol has been developed and published to date. The PPI panel were consulted for their contribution to the research question prior to publication of the protocol. Implementation of the search strategy commenced in the last week of January 2025. It is anticipated that the review will be completed and submitted for publication by the end of 2025.

## Ethics and consent

Ethical approval and consent were not required.

## Data Availability

No data are associated with this article. Open Science Framework (OSF): What barriers and facilitators to self-management are experienced by mothers who wish to breastfeed but require concurrent pharmacotherapy in the first two years postpartum? A mixed-methods systematic review protocol: Extended data. https://doi.org/10.17605/OSF.IO/DUFMG ^39^ This project contains the following extended data: Search strategy for six electronic databases – CINAHL, EMBASE, Medline (Ovid), PsycINFO, Maternity and Infant Care, and Global Index Medicus. Draft data extraction form Eligibility criteria table PRISMA-P checklist PRISMA flow diagram to date Open Science Framework: PRISMA-P checklist for ‘What barriers and facilitators to self-management are experienced by mothers who wish to breastfeed but require concurrent pharmacotherapy in the first two years postpartum? A mixed-methods systematic review protocol.’ https://doi.org/10.17605/OSF.IO/DUFMG ^39^. Data are available under the terms of the Creative Commons Zero 1.0 Universal licence.

## Software availability

The research team will use Covidence and Endnote proprietary software. Alternative tools which are free and open access include Rayyan for screening of papers and Zotero for reference management.

## References

[1] World Health Organization, United Nation's Children's Fund (UNICEF): Global breastfeeding scorecard.Geneva, Switzerland: World Health Organization,2023; [cited 2025 Feb 05]. Reference Source

[2] Shrimpton R : Continued breastfeeding for healthy growth and development of children.USA: World Health Organization,2017; [cited 2025 Feb 05]. Reference Source

[3] United Nation's Children's Fund (UNICEF) Ireland: Action needed to improve Ireland's low breastfeeding rates.UNICEF,2024; [cited 2024 Oct 16]. Reference Source

[4] World Health Organization: World health statistics 2014.Geneva, Switzerland: World Health Organization,2014; [cited 2024 Oct 16]. Reference Source

[5] Odom EC Li RW Scanlon KS : Reasons for earlier than desired cessation of breastfeeding. *Pediatrics.* 2013;131(3):E726–E732. 10.1542/peds.2012-1295 23420922 PMC4861949

[6] Declercq ER Sakala C Corry MP : New mothers speak out: national survey results highlight women’s postpartum experiences.New York: Childbirth Connection,2008. Reference Source

[7] Li RW Fein SB Chen J : Why mothers stop breastfeeding: mothers' self-reported reasons for stopping during the first year. *Pediatrics.* 2008;122 Suppl 2(Supplement 2):S69–S76. 10.1542/peds.2008-1315i 18829834

[8] Daly D Higgins A Hannon S : Trajectories of postpartum recovery: what is known and not known. *Clin Obstet Gynecol.* 2022;65(3):594–610. 10.1097/GRF.0000000000000726 35797600

[9] Hannon S Gartland D Higgins A : Physical health and comorbid anxiety and depression across the first year postpartum in Ireland (MAMMI study): a longitudinal population-based study. *J Affect Disord.* 2023;328:228–237. 10.1016/j.jad.2023.02.056 36801420

[10] Schirm E Schwagermann MP Tobi H : Drug use during breastfeeding. A survey from the Netherlands. *Eur J Clin Nutr.* 2004;58(2):386–390. 10.1038/sj.ejcn.1601799 14749761

[11] Al-Sawalha NA Tahaineh L Sawalha A : Medication use in breastfeeding women: a national study. *Breastfeed Med.* 2016;11(7):386–91. 10.1089/bfm.2016.0044 27548275

[12] Stultz EE Stokes JL Shaffer ML : Extent of medication use in breastfeeding women. *Breastfeed Med.* 2007;2(3):145–151. 10.1089/bfm.2007.0010 17903100

[13] de Waard M Blomjous BS Hol ML : Medication use during pregnancy and lactation in a Dutch population. *J Hum Lact.* 2019;35(1):154–164. 10.1177/0890334418775630 29969343

[14] Bakker MK Jentink J Vroom F : Maternal medicine: drug prescription patterns before, during and after pregnancy for chronic, occasional and pregnancy‐related drugs in the Netherlands. *BJOG.* 2006;113(5):559–568. 10.1111/j.1471-0528.2006.00927.x 16637899

[15] Scime NV Metcalfe A Nettel-Aguirre A : Association of postpartum medication practices with early breastfeeding cessation among mothers with chronic conditions: a prospective cohort study. *Acta Obstet Gynecol Scand.* 2023;102(4):420–429. 10.1111/aogs.14516 36707933 PMC10008275

[16] Julsgaard M Nørgaard M Hvas CL : Self-reported adherence to medical treatment, breastfeeding behaviour, and disease activity during the postpartum period in women with Crohn’s disease. *Scand J Gastroenterol.* 2014;49(8):958–966. 10.3109/00365521.2014.920913 24897523

[17] Spiesser-Robelet L Maurice A Gagnayre R : Understanding breastfeeding women’s behaviors toward medication: healthcare professionals’ viewpoint. *J Hum Lact.* 2019;35(1):137–153. 10.1177/0890334418771294 29727253

[18] Spiesser-Robelet L Brunie V de Andrade V : Knowledge, representations, attitudes, and behaviors of women faced with taking medications while breastfeeding: a scoping review. *J Hum Lact.* 2017;33(1):98–114. 10.1177/0890334416679383 28027444

[19] Saha MR Ryan K Amir LH : Postpartum women’s use of medicines and breastfeeding practices: a systematic review. *Int Breastfeed J.* 2015;10: 28. 10.1186/s13006-015-0053-6 26516340 PMC4625926

[20] Sachs HC , Committee On Drugs: The transfer of drugs and therapeutics into human breast milk: an update on selected topics. *Pediatrics.* 2013;132(3):e796–e809. 10.1542/peds.2013-1985 23979084

[21] Berlin CM Jr van den Anker JN : Safety during breastfeeding: drugs, foods, environmental chemicals, and maternal infections. *Semin Fetal Neonatal Med.* 2013;18(1):13–18. 10.1016/j.siny.2012.09.003 23131768

[22] Davanzo R Bua J De Cunto A : Advising mothers on the use of medications during breastfeeding: a need for a positive attitude. *J Hum Lact.* 2016;32(1):15–19. 10.1177/0890334415595513 26173811

[23] Anderson PO Pochop SL Manoguerra AS : Adverse drug reactions in breastfed infants: less than imagined. *Clin Pediatr (Phila).* 2003;42(4):325–340. 10.1177/000992280304200405 12800727

[24] Nordeng H Havnen GC Spigset O : Drug use and breastfeeding. *Tidsskr Nor Laegeforen.* 2012;132(9):1089–1093. 10.4045/tidsskr.11.1104 22614307

[25] McDonald K Amir LH Davey MA : Maternal bodies and medicines: a commentary on risk and decision-making of pregnant and breastfeeding women and health professionals. *BMC Public Health.* 2011;11 Suppl 5(Suppl 5): S5. 10.1186/1471-2458-11-S5-S5 22168473 PMC3247028

[26] Amir LH Pirotta MV Raval M : Breastfeeding--evidence based guidelines for the use of medicines. *Aust Fam Physician.* 2011;40(9):684–690. 21894275

[27] Jones W : Breastfeeding and medication.2nd. ed. New York: Routledge,2018. Reference Source

[28] Marshall AM Nommsen-Rivers LA Hernandez LL : Serotonin transport and metabolism in the mammary gland modulates secretory activation and involution. *J Clin Endocrinol Metab.* 2010;95(2):837–46. 10.1210/jc.2009-1575 19965920 PMC2840848

[29] Cadwell K Brimdyr K : Intrapartum administration of synthetic oxytocin and downstream effects on breastfeeding: elucidating physiologic pathways. *Ann Nurs Res Pract.* 2017;2(3):1024. Reference Source

[30] Erickson EN Emeis CL : Breastfeeding outcomes after oxytocin use during childbirth: an integrative review. *J Midwifery Womens Health.* 2017;62(4):397–417. 10.1111/jmwh.12601 28759177

[31] Zhou Y Liu W Xu Y : Effects of different doses of synthetic oxytocin on neonatal instinctive behaviors and breastfeeding. *Sci Rep.* 2022;12(1): 16434. 10.1038/s41598-022-20770-y 36180494 PMC9525660

[32] Bailey SC Oramasionwu CU Wolf MS : Rethinking adherence: a health literacy-informed model of medication self-management. *J Health Commun.* 2013;18 Suppl 1(Suppl 1):20–30. 10.1080/10810730.2013.825672 24093342 PMC3814610

[33] Holden RJ Carayon P Gurses AP : SEIPS 2.0: a human factors framework for studying and improving the work of healthcare professionals and patients. *Ergonomics.* 2013;56(11):1669–1686. 10.1080/00140139.2013.838643 24088063 PMC3835697

[34] Hussainy SY Dermele N : Knowledge, attitudes and practices of health professionals and women towards medication use in breastfeeding: a review. *Int Breastfeed J.* 2011;6: 11. 10.1186/1746-4358-6-11 21867562 PMC3180355

[35] Pilgrim R Kwok M May A : The effect of medication use on breastfeeding continuation: a systematic review with narrative synthesis. *Int Breastfeed J.* 2025;20(1): 59. 10.1186/s13006-025-00756-y 40760660 PMC12320353

[36] Holden RJ Carayon P : SEIPS 101 and seven simple SEIPS tools. *BMJ Qual Saf.* 2021;30(11):901–910. 10.1136/bmjqs-2020-012538 34039748 PMC8543199

[37] Carayon P Wooldridge A Hoonakker P : SEIPS 3.0: human-centered design of the patient journey for patient safety. *Appl Ergon.* 2020;84: 103033. 10.1016/j.apergo.2019.103033 31987516 PMC7152782

[38] Moher D Shamseer L Clarke M : Preferred Reporting Items for Systematic review and Meta-Analysis Protocols (PRISMA-P) 2015 statement. *Syst Rev.* 2015;4(1): 1. 10.1186/2046-4053-4-1 25554246 PMC4320440

[39] Hackett L D'Arcy DM O'Connell J : What barriers and facilitators to self-management are experienced by mothers who wish to breastfeed but require concurrent pharmacotherapy in the first two years postpartum? A mixed-methods systematic review protocol: extended data. Open Science Framework.2025; [updated 2025 Nov 18; cited 2025 Nov 18]. https://osf.io/dufmg/ 10.12688/hrbopenres.14100.3 PMC12789855 41523928

[40] Stern C Lizarondo L Carrier J : Methodological guidance for the conduct of mixed methods systematic reviews. *JBI Evid Implement.* 2021;19(2):120–129. 10.1097/XEB.0000000000000282 34061049

[41] Khan KS Kunz R Kleijnen J : Systematic reviews to support evidence-based medicine: how to review and apply findings of healthcare research.London: Royal Society of Medicine Press,2003. Reference Source

[42] Page MJ McKenzie JE Bossuyt PM : The PRISMA 2020 statement: an updated guideline for reporting systematic reviews. *BMJ.* 2021;372: n71. 10.1136/bmj.n71 33782057 PMC8005924

[43] Hong QN Pluye P Fàbregues S : Mixed Methods Appraisal Tool (MMAT) version 2018: user guide.McGill,2018; [updated 2018 Aug 1; cited 2024 Oct 16]. Reference Source

[44] Pollock A Campbell P Struthers C : Development of the ACTIVE framework to describe stakeholder involvement in systematic reviews. *J Health Serv Res Policy.* 2019;24(4):245–255. 10.1177/1355819619841647 30997859

