# Supplementary material for: What barriers and facilitators to self-management are experienced by mothers who wish to breastfeed but require concurrent pharmacotherapy in the first two years postpartum? A mixed-methods systematic review protocol
Source: HRB Open Res. 2026 Feb 17;8:53. Originally published 2025 Apr 10. [Version 3] doi: 10.12688/hrbopenres.14100.3 (PMC12789855; doi:10.12688/hrbopenres.14100.3)
Supplement: Peer review history

# Reviewer report: Reviewer response for version 3

**DOI:** 10.21956/hrbopenres.15802.r53602

**Published:** 2026-03-02

**Authors:** Sue Jordan

**Affiliations:** 1 Swansea University, Swansea, UK

**Protocol for systematic review on barriers to breastfeeding whilst taking medicines**

 Thank you for the opportunity to review this protocol. Women’s knowledge and attitudes are important.

 The main concern with this review protocol relates to the omission of probably the most critical factor determining the initiation, continuation and success of breastfeeding: the biological effects of medicines on the physiology of lactation and infant behaviour. For example, the biological effects of serotonergic agents (including many antidepressants) on lactation are reported in both biomedical [1,2] and pharmaco-epidemiological research [3,4], independently of the underlying condition [5]. Women prescribed antidepressants have more difficulty in breastfeeding, with delays of ~15 hours reported [1], and need additional support [5,6,7]. Many medicines impair lactation and breastfeeding, including amphetamines, oestrogens, ergotamine derivatives, aripiprazole, promethazine, diuretics, opioids and alcohol. Polypharmacy and recreational drug use are important considerations for any work on breastfeeding [5], and should be included in all analyses.

 Healthcare professionals and families may also consider the need for additional monitoring of infants, particularly those born preterm, exposed to some medicines via breastmilk, including psychotropic medicines and sedatives [8], and the impact on services [5].

**References**

 1.Marshall AM, Nommsen-Rivers LA, Hernandez LL, Dewey KG, Chantry CJ, Gregerson KA, Horseman ND. Serotonin transport and metabolism in the mammary gland modulates secretory activation and involution. J Clin Endocrinol Metab. 2010 Feb;95(2):837-46. doi: 10.1210/jc.2009-1575. Epub 2009 Dec 4. PMID: 19965920; PMCID: PMC2840848.

 2. Nyárády, K., R. Turai, and S. Funke, *Effects of perinatal factors on sirtuin 3, 8-hydroxy-2′- deoxyguanosine, brain-derived neurotrophic factor and serotonin in cord blood and early breast milk: an observational study.* Int Breastfeed J, 2020. 15: p. 57.

 3. Gorman, J. R.-.-., et al. (2012). "Breastfeeding among women exposed to antidepressants during pregnancy." J Hum Lact 28(2): 181-188.

 4. Grzeskowiak LE, Saha MR, Nordeng H, Ystrom E, Amir LH. Perinatal antidepressant use and breastfeeding outcomes: Findings from the Norwegian Mother, Father and Child Cohort Study. Acta Obstet Gynecol Scand. 2022 Feb 16. doi: 10.1111/aogs.14324.

 5. Jordan S, Davies GI, Thayer DS., Tucker D., Humphreys I. (2019) Antidepressant prescriptions, discontinuation, depression and perinatal outcomes, including breastfeeding: a population cohort analysis. Plos One, 14(11):  e0225133. https://doi.org/10.1371/journal.pone.0225133

 6. LactMed, Drugs and Lactation Database (LactMed®) [Internet]. Bethesda (MD): National Institute of Child Health and Human Development; 2006–. Sertraline. 2025 Feb 15. PMID: 30000250. Sertraline - Drugs and Lactation Database (LactMed®) - NCBI Bookshelf

 7. Jordan S, Komninou S, Lopez Leon S (2023) Where are the data linking infant outcomes, breastfeeding and medicine exposure? A systematic scoping review. PLOS ONE 18(4): e0284128. https://doi.org/10.1371/journal.pone.0284128

 8. BNF/ Medicines Complete (2025) Psychotropic drug directory MedicinesComplete — CONTENT > Psychotropic Drug Directory > Problem areas: 3.9 Perinatal

Is the study design appropriate for the research question?

Partly

Is the rationale for, and objectives of, the study clearly described?

Partly

Are sufficient details of the methods provided to allow replication by others?

No

Are the datasets clearly presented in a useable and accessible format?

Not applicable

Reviewer Expertise:

NA

I confirm that I have read this submission and believe that I have an appropriate level of expertise to confirm that it is of an acceptable scientific standard, however I have significant reservations, as outlined above.

## References

[1] : Serotonin Transport and Metabolism in the Mammary Gland Modulates Secretory Activation and Involution. *The Journal of Clinical Endocrinology & Metabolism* .2010;95(2) : 10.1210/jc.2009-1575 837-846 10.1210/jc.2009-1575 PMC284084819965920

[2] : Effects of perinatal factors on sirtuin 3, 8-hydroxy-2′- deoxyguanosine, brain-derived neurotrophic factor and serotonin in cord blood and early breast milk: an observational study. *International Breastfeeding Journal* .2020;15(1) : 10.1186/s13006-020-00301-z 10.1186/s13006-020-00301-z 32552911 PMC7302386

[3] : Breastfeeding among Women Exposed to Antidepressants during Pregnancy. *Journal of Human Lactation* .2012;28(2) : 10.1177/0890334411429782 181-188 10.1177/0890334411429782 22344850

[4] : Perinatal antidepressant use and breastfeeding outcomes: Findings from the Norwegian Mother, Father and Child Cohort Study. *Acta Obstetricia et Gynecologica Scandinavica* .2022;101(3) : 10.1111/aogs.14324 344-354 10.1111/aogs.14324 PMC956455635170756

[5] : Antidepressant prescriptions, discontinuation, depression and perinatal outcomes, including breastfeeding: A population cohort analysis. *PLOS ONE* .2019;14(11) : 10.1371/journal.pone.0225133 10.1371/journal.pone.0225133 PMC686044031738813

[6] : Where are the data linking infant outcomes, breastfeeding and medicine exposure? A systematic scoping review. *PLOS ONE* .2023;18(4) : 10.1371/journal.pone.0284128 10.1371/journal.pone.0284128 PMC1013255237099508

# Reviewer report: Reviewer response for version 2

**DOI:** 10.21956/hrbopenres.15735.r52125

**Published:** 2026-01-10

**Authors:** Essi Whaites Heinonen

**Affiliations:** 1 Karolinska Institutet, Stockholm, Stockholm County, Sweden

Thank you for inviting me to review this study protocol! But most of all, thank you for shedding light on this very important research question, in a gravely underexplored research area. Due to improved treatment of chronic conditions, more women using chronic medications become pregnant and give birth to healthy thriving infants, eager to breastfeed. It is our responsibility as health care providers and researchers to guide the women in need of medications through the perinatal period and answer their questions on the breastfeeding safety of their treatment. Current evidence shows that mothers treated with chronic medications are less likely to follow the breastfeeding recommendations, but as this planned study points out, the reasons behind this are largely unstudied. For us as a research community it makes a great difference to understand, whether these mothers choose against breastfeeding due to the disease severity, or due to safety concerns of the medication for the breastfed infant when safety data is lacking. In my opinion, this planned study is welcomed and seems adequately planned to increase our understanding the barriers and facilitators of breastfeeding in regard to maternal medications, and to highlight further research questions in order to advance this field of research, and the healthcare for these women and their newborn infants. I don´t have personal experience in performing mixed methods analyses, but considering this has been recommended by previous research, it seems reasonable to try and perform it. The selected PEO, the described method and the chosen software also seem adequate to creating robust evidence out of existing data.

 Minor comments:

 Comment 1. In the background, please consider lifting the markedly lower breastfeeding rates in mothers chronically using antirheumatics (1), where we currently do not know whether these mothers choose against breastfeeding due to concerns regarding medication safety or the severity of the underlying rheumatic or inflammatory bowel disorder.

 Comment 2. Regarding the time frame, you write that the results are aimed to be submitted by the end of 2025. Please revise this, if necessary.

Is the study design appropriate for the research question?

Yes

Is the rationale for, and objectives of, the study clearly described?

Yes

Are sufficient details of the methods provided to allow replication by others?

Yes

Are the datasets clearly presented in a useable and accessible format?

Yes

Reviewer Expertise:

Pediatrics, perinatal pharmacoepidemiology

I confirm that I have read this submission and believe that I have an appropriate level of expertise to confirm that it is of an acceptable scientific standard.

## References

[1] : Lower Adherence to Breastfeeding Recommendations in Mothers Treated With Antirheumatic and Antidepressant Medications. *Journal of Human Lactation* .2025;41(3) : 10.1177/08903344251337384 412-422 10.1177/08903344251337384 40432295 PMC12238658

# Response

**Published:** 2026-01-29

**Authors:** Lucy Hackett

**Affiliations:** Trinity College Dublin School of Pharmacy and Pharmaceutical Sciences, Dublin, Leinster, Ireland

Many thanks for taking the time to review our protocol and offer your expertise.

Reviewer comment 1:

 In the background, please consider lifting the markedly lower breastfeeding rates in mothers chronically using antirheumatics (1), where we currently do not know whether these mothers choose against breastfeeding due to concerns regarding medication safety or the severity of the underlying rheumatic or inflammatory bowel disorder.

Response:

 I hope I have understood and interpreted this suggestion correctly. It is important to acknowledge that in much of the literature where lower breastfeeding rates are noted in women who require pharmacotherapy compared to their counterparts who do not, it is not always known whether this is due to medication safety concerns, or because of the underlying condition being treated. Should it be possible during the review process, these differences will be noted to enhance understanding of women’s apparent decline in breastfeeding following exposure to medication, and the effect of the confounding variable – that is, the disease or condition itself - on breastfeeding. It may be possible to further explore the relationships between these variables through this review; for example, the possibility that medication discontinuation is also associated with shorter breastfeeding duration ^(1)^ may be a result of the untreated disease having an impact upon breastfeeding success. Investigating women’s self-management decisions in these situations will help to understand this current gap in the literature. The following has been added to the background section of this protocol to reflect this (changes in bold):

 ‘ *Medication use for the management of chronic conditions has been shown to decline in pregnancy and remains low in the months following, most likely due to breastfeeding* *^14^* *. This may suggest that some women will prioritise infant feeding over their own health if they perceive that these goals are incompatible. It has been reported that women who discontinue pre-existing medications while breastfeeding have shorter breastfeeding duration and are less likely to meet their personal breastfeeding goals than women who breastfeed while continuing effective medications* *^15^* *. This is a highly unfavourable situation, as women may suffer morbidity due to medication discontinuation, while also not benefiting from the protective effects of continued breastfeeding for themselves or their infants. **It is unknown if reduced breastfeeding rates in these scenarios are the result of medication safety concerns or effects of the underlying condition itself upon breastfeeding – as has been noted briefly in the literature ^15,16^. It is hoped that this review will facilitate further exploration of the nuanced relationship between these variables.***

Reviewer comment 2:

 Regarding the time frame, you write that the results are aimed to be submitted by the end of 2025. Please revise this, if necessary.

Response:

 Many thanks for highlighting this. This has been removed from the manuscript and has been revised to the end of 2026:

*‘The review protocol has been developed and published to date. The PPI panel were consulted for their contribution to the research question prior to publication of the protocol. Implementation of the search strategy commenced in the last week of January 2025. It is anticipated that the review will be completed and submitted for publication **in 2026**.’*

References:

1. Scime NV, Metcalfe A, Nettel-Aguirre A, Tough SC, Chaput KH. Association of postpartum medication practices with early breastfeeding cessation among mothers with chronic conditions: A prospective cohort study. Acta Obstet Gyn Scan. 2023;102(4):420-9.

# Reviewer report: Reviewer response for version 2

**DOI:** 10.21956/hrbopenres.15735.r51702

**Published:** 2025-12-31

**Authors:** Ana Veronica Scotta

**Affiliations:** 1 Universidad Nacional de Cordoba, Córdoba, Cordoba, Argentina

Thank you again for the opportunity to review this manuscript.

 Most of the review issues have been addressed by the authors. However, there are still areas that I believe require further clarification:

 A table has been added as supplementary material (reference 39), detailing all queried databases, the search strategy for each, and the results. However, some databases show results in the millions, which is excessively high for screening and could indicate the need to revise the search strategy, or add time limits to the search.

 Additionally, the number of search results reported in the aforementioned table does not align with the numbers presented in the PRISMA flowchart provided in the same supplementary material. These discrepancies should be addressed and corrected.

 Similarly, the authors report obtaining thousands of references from the preliminary gray literature search, and cite this as one of the reasons for not including these sources. The manuscript should include a clear justification for excluding gray literature, as its inclusion is considered a recommended practice in evidence synthesis.

Is the study design appropriate for the research question?

Yes

Is the rationale for, and objectives of, the study clearly described?

Yes

Are sufficient details of the methods provided to allow replication by others?

Partly

Are the datasets clearly presented in a useable and accessible format?

Not applicable

Reviewer Expertise:

Maternal health, breastfeeding determinants, epidemiology.

I confirm that I have read this submission and believe that I have an appropriate level of expertise to confirm that it is of an acceptable scientific standard, however I have significant reservations, as outlined above.

# Response

**Published:** 2026-01-29

**Authors:** Lucy Hackett

**Affiliations:** Trinity College Dublin School of Pharmacy and Pharmaceutical Sciences, Dublin, Leinster, Ireland

Reviewer comment 1:

 A table has been added as supplementary material (reference 39), detailing all queried databases, the search strategy for each, and the results. However, some databases show results in the millions, which is excessively high for screening and could indicate the need to revise the search strategy, or add time limits to the search.

Response:

 Many thanks for taking the time to review the changes to our protocol. In the table in question, any numbers in the millions refer to broad search terms (such as the search string for ‘medication’ and synonyms), which is not unexpected. However, when these broad terms are combined to reflect our focused research question, the search results produce large but manageable numbers for screening from each database. This search strategy was the result of many iterations of similar searches to find the most effective strategy in terms of sensitivity and specificity, with input from a subject librarian. Due to the significant number of papers which now appear relevant through the process of dual screening, limitations on the year of publication are currently being considered to facilitate a focused, up-to-date and timely systematic review.  

Reviewer comment 2:

 Additionally, the number of search results reported in the aforementioned table does not align with the numbers presented in the PRISMA flowchart provided in the same supplementary material. These discrepancies should be addressed and corrected.

Response:

 Apologies for this. The discrepancy in question appears to be attributable to the Global Index Medicus database – which was included at a later date – not having been added to the PRISMA flow diagram during the previous revision of the protocol. All other numbers align with the results retrieved from database searches.  This issue has now been rectified in the supplementary material.

Reviewer comment 3:

 Similarly, the authors report obtaining thousands of references from the preliminary gray literature search, and cite this as one of the reasons for not including these sources. The manuscript should include a clear justification for excluding gray literature, as its inclusion is considered a recommended practice in evidence synthesis.

Response:

 Grey literature will not be included in this review considering the impractically large numbers of results returned from scoping searches. Additionally, there is an abundance of published, peer reviewed literature on this topic – as reflected in the search strategy, dual screening of papers at both abstract and full text stages, and in the number of papers now being considered for inclusion in the review. Therefore, there was deemed to be sufficient data available from published, peer reviewed literature, and our review will remain based on this literature. The following has been added to the manuscript to reflect this (changes in bold):

*‘Six electronic databases will be systematically searched: CINAHL (Cumulative Index to Nursing and Allied Health Literature), EMBASE (Excerpta Medica Database), PsycINFO, Medline (Ovid), Maternity and Infant Care, and Global Index Medicus. Databases will be searched using terms related to medication, breastfeeding, women, experience, barriers, and facilitators. Searches will be limited to peer reviewed, empirical studies published in the English language, and not limited by year. Other literature reviews will be excluded. **Grey literature will not be included in this review as preliminary searches of available grey literature sources returned impractical numbers of results for screening. Moreover, the reports retrieved from focused searches of bibliographic databases reflect an abundance of relevant published, peer-reviewed literature on this topic. **The search strategies used for all six databases are available ^40^* *. Additionally, literature will be hand-searched from identified pertinent systematic reviews or included studies by checking for relevant cited or citing papers.*

# Reviewer report: Reviewer response for version 1

**DOI:** 10.21956/hrbopenres.15493.r47082

**Published:** 2025-06-06

**Authors:** Sue Jordan

**Affiliations:** 1 Swansea University, Swansea, UK

**Protocol for systematic review on barriers to breastfeeding whilst taking medicines**

 Thank you for the opportunity to review this protocol. Women’s knowledge and attitudes are important. 

 The main concern with this review protocol relates to the omission of probably the most critical factor determining the initiation, continuation and success of breastfeeding: the biological effects of medicines on the physiology of lactation and infant behaviour. For example, the biological effects of serotonergic agents (including many antidepressants) on lactation are reported in both biomedical [1,2] and pharmaco-epidemiological research [3,4], independently of the underlying condition [5]. Women prescribed antidepressants have more difficulty in breastfeeding, with delays of ~15 hours reported [1], and need additional support [5,6,7]. Many medicines impair lactation and breastfeeding, including amphetamines, oestrogens, ergotamine derivatives, aripiprazole, promethazine, diuretics, opioids and alcohol. Polypharmacy and recreational drug use are important considerations for any work on breastfeeding [5] and should be included in all analyses. 

 Healthcare professionals and families may also consider the need for additional monitoring of infants, particularly those born preterm, exposed to some medicines via breastmilk, including psychotropic medicines and sedatives [8], and the impact on services [5]. 

 It should be acknowledged that, for some medicines, such as clozapine or mycophenolate mofetil, the benefits of breastfeeding may not outweigh possible adverse effects of medicines ingested via breastmilk; this is reflected in manufacturers’ advice [8].  

 It would have been helpful to view the search strategy alongside the methods. There are also questions regarding the selection and deployment of ‘risk of bias’ tools, and the PICO format without identified comparators. The authors should clarify any funding from the pharmaceutical industry, making it even more critical that a holistic approach be taken to the relationship between prescribed medicines and breastfeeding. I hope this review will help the team contextualise their work.

**References**

 1.Marshall AM, Nommsen-Rivers LA, Hernandez LL, Dewey KG, Chantry CJ, Gregerson KA, Horseman ND. 2010 (Ref 1)

 2. Nyárády, K., R. Turai, and S. Funke 2020 (Ref 2)

 3. Gorman, J. R., et al. 2012 (Ref 3)

 4. Grzeskowiak LE, Saha MR, Nordeng H, Ystrom E, Amir LH. (Ref 4)

 5. Jordan S, Davies GI, Thayer DS., Tucker D., Humphreys I. 2019 (Ref 5)

 6. LactMed, Drugs and Lactation Database (LactMed®) Bethesda (MD): 2025 (Ref 6)

 7. Jordan S, Komninou S, Lopez Leon S 2023 (Ref 7)

 8. BNF/ Medicines Complete 2025 Psychotropic drug directory MedicinesComplete — CONTENT > Psychotropic Drug Directory > Problem areas: 3.9 Perinatal https://www.pharmaceuticalpress.com/products/psychotropic-drug-directory/

Is the study design appropriate for the research question?

Partly

Is the rationale for, and objectives of, the study clearly described?

Partly

Are sufficient details of the methods provided to allow replication by others?

No

Are the datasets clearly presented in a useable and accessible format?

Not applicable

Reviewer Expertise:

Medicines management, breastfeeding, pregnancy, polypharmacy.

I confirm that I have read this submission and believe that I have an appropriate level of expertise to confirm that it is of an acceptable scientific standard, however I have significant reservations, as outlined above.

## References

[1] : Serotonin transport and metabolism in the mammary gland modulates secretory activation and involution. *J Clin Endocrinol Metab* .2010;95(2) : 10.1210/jc.2009-1575 837-46 10.1210/jc.2009-1575 19965920 PMC2840848

[2] : Effects of perinatal factors on sirtuin 3, 8-hydroxy-2'- deoxyguanosine, brain-derived neurotrophic factor and serotonin in cord blood and early breast milk: an observational study. *Int Breastfeed J* .2020;15(1) : 10.1186/s13006-020-00301-z 57 10.1186/s13006-020-00301-z 32552911 PMC7302386

[3] : Breastfeeding among women exposed to antidepressants during pregnancy. *J Hum Lact* .2012;28(2) : 10.1177/0890334411429782 181-8 10.1177/0890334411429782 22344850

[4] : Perinatal antidepressant use and breastfeeding outcomes: Findings from the Norwegian Mother, Father and Child Cohort Study. *Acta Obstet Gynecol Scand* .2022;101(3) : 10.1111/aogs.14324 344-354 10.1111/aogs.14324 35170756 PMC9564556

[5] : Antidepressant prescriptions, discontinuation, depression and perinatal outcomes, including breastfeeding: A population cohort analysis. *PLoS One* .2019;14(11) : 10.1371/journal.pone.0225133 e0225133 10.1371/journal.pone.0225133 31738813 PMC6860440

[6] Sertraline.2006; 30000250

[7] : Where are the data linking infant outcomes, breastfeeding and medicine exposure? A systematic scoping review. *PLoS One* .2023;18(4) : 10.1371/journal.pone.0284128 e0284128 10.1371/journal.pone.0284128 37099508 PMC10132552

# Response

**Published:** 2025-11-19

**Authors:** Lucy Hackett

**Affiliations:** Trinity College Dublin School of Pharmacy and Pharmaceutical Sciences, Dublin, Leinster, Ireland

Many thanks for taking the time to review this protocol and for providing valuable feedback to improve the quality of our systematic review. We have revised our protocol following this and have individually addressed each comment below. Changes to the text are outlined in bold.

Reviewer comment 1:

 Thank you for the opportunity to review this protocol. Women’s knowledge and attitudes are important. 

**Response:**

 Thank you for taking the time to review our systematic review protocol.

Reviewer comment 2:

 The main concern with this review protocol relates to the omission of probably the most critical factor determining the initiation, continuation and success of breastfeeding: the biological effects of medicines on the physiology of lactation and infant behaviour. For example, the biological effects of serotonergic agents (including many antidepressants) on lactation are reported in both biomedical [1,2] and pharmaco-epidemiological research [3,4], independently of the underlying condition [5]. Women prescribed antidepressants have more difficulty in breastfeeding, with delays of ~15 hours reported [1], and need additional support [5,6,7]. Many medicines impair lactation and breastfeeding, including amphetamines, oestrogens, ergotamine derivatives, aripiprazole, promethazine, diuretics, opioids and alcohol.

**Response:**

 Many thanks for highlighting this important point. While the authors acknowledge that the biological effects of medication on the physiology of lactation and infant behaviour is an important factor in breastfeeding, this review will focus on women’s experiences of the barriers and facilitators to self-management. Therefore, if the literature reveals that women have reported the biological effects of medication as an important factor, it will of course be included in this review.

 The following paragraph has been added to the background to provide further clarification:

***‘Furthermore, there is a potential for some medications to have physiological effects on lactation and infant behaviour. This has been noted in the literature in relation to antidepressants*** ***(28)*** ***and intrapartum synthetic*** ***oxytocin*** ***(29-31)*** ***, for example. Little is known about whether and how this may impact medication self-management and women’s choices when pharmacotherapy and breastfeeding coincide. The effects of medication on lactational physiology and infant behaviour may be one consideration among many for these women.’***

Reviewer comment 3:

 Polypharmacy and recreational drug use are important considerations for any work on breastfeeding [5] and should be included in all analyses. 

**Response:**

 Polypharmacy is indeed an important consideration in this area. We have revised the last sentence of the data synthesis section to reflect this:

*‘If data from the literature permits, analysis will differentiate between occasional and chronic medication needs, **as well as polypharmacy and single medication use,** **and how each of these are associated with breastfeeding. Other subgroups, such as infant age categories, will also be considered if possible.’***

 Recreational or illicit drug use is not the focus of this review; rather, eligible studies reporting women’s experiences relating to authorised medications (prescription-only, over-the-counter medication or dietary supplements) will be included for analysis. While women’s self-management in the context of recreational drug use is important, we believe it merits a separate review. The focus on authorised medications will allow unique insight into women’s experiences when trying to navigate the health system and the specific use of authorised medications in the context of breastfeeding. Authorised medications will include those prescribed for opioid substitution therapy.

Reviewer comment 4:

 Healthcare professionals and families may also consider the need for additional monitoring of infants, particularly those born preterm, exposed to some medicines via breastmilk, including psychotropic medicines and sedatives [8], and the impact on services [5]. 

**Response:**

 This is an important point and may be a component of the patient work and self-management activities for women taking medications that may pass to their infant through breastmilk. If such data is reported in included studies, it will be extracted and analysed.

Reviewer comment 5:

 It should be acknowledged that, for some medicines, such as clozapine or mycophenolate mofetil, the benefits of breastfeeding may not outweigh possible adverse effects of medicines ingested via breastmilk; this is reflected in manufacturers’ advice [8].

**Response:**

 This is reflected in the following sentence in the background section:

*‘While a small number of medications are accepted as contraindicated during breastfeeding *(20-22) *, many medications needed during this period may be considered safe or to have a safe alternative; there are a relatively small number of adverse events reported in breastfed infants whose mothers have taken medication* (23) *and the amount of drug transferred to the infant via the breastmilk is usually too small to exert any effect* (24) *. However, deciphering which medicines are safe may be a difficult task.* *’*

Reviewer comment 6:

 It would have been helpful to view the search strategy alongside the methods.

**Response:**

 The search strategy for one electronic database (Cumulative Index to Nursing Allied Health Literature [CINAHL]) was made available on the Open Science Framework (OSF) online repository and referenced within the text. This search strategy was replicated across all other databases mentioned (EMBASE, PsycINFO, Medline Ovid, Maternity and Infant Care, Global Index Medicus). For additional transparency, the search strategy for all electronic databases has now been added to the online OSF repository (1) and referenced in the text.

 The following sentence has been updated in the ‘search strategy’ section to reflect this:

**‘ *The search strategies used for all six databases are available (39)’***

Reviewer comment 7:

 There are also questions regarding the selection and deployment of ‘risk of bias’ tools, and the PICO format without identified comparators.

**Response:**

 Please see the responses to Reviewer One, Ana Veronica Scotta, in relation to the PICO question framework and quality assessment tools.

Reviewer comment 8:

 The authors should clarify any funding from the pharmaceutical industry, making it even more critical that a holistic approach be taken to the relationship between prescribed medicines and breastfeeding.

**Response:**

 The sentence below from the protocol clarifies the funding for this research. The author acknowledges the support of the Panoz Pharmaceutical Innovation PhD Scholarship, awarded by the School of Pharmacy and Pharmaceutical Sciences, Trinity College Dublin. 

*‘This research was funded by the Panoz Pharmaceutical Innovation PhD Scholarship at Trinity College Dublin. The funders had no role in study design, data collection and analysis, decision to publish, or preparation of the manuscript.’*

 This scholarship was made possible through a generous bequest from the estate of Dr. Don Panoz. It covers PhD fees, a stipend for the PhD researcher, provision for the purchase of a laptop and support for research travel costs. There is no further funding for this research.

References:

**1.** Hackett L, D'Arcy DM, O'Connell J, Cromie S, Daly D, Grimes T. What barriers and facilitators to self-management are experienced by mothers who wish to breastfeed but require concurrent pharmacotherapy in the first two years postpartum? A mixed-methods systematic review protocol: extended data. Open Science Framework; 2025; [updated 2025 Nov 18; cited 2025 Nov 18]. https://osf.io/dufmg/

# Reviewer report: Reviewer response for version 1

**DOI:** 10.21956/hrbopenres.15493.r47232

**Published:** 2025-06-03

**Authors:** Ana Veronica Scotta

**Affiliations:** 1 Universidad Nacional de Cordoba, Córdoba, Cordoba, Argentina

This mixed-methods systematic review protocol aims to explore the barriers and facilitators to self-management for women who consider breastfeeding during pharmacotherapy within the first two years postpartum. The rationale for this review is sufficiently detailed, as well as the gap in the evidence it aims to address.

 Background: When mentioning two previous reviews about the topic of breastfeeding and pharmacotherapy, I advise the authors to mention that none of these articles follow a systematic review methodology. Therefore, the proposed review would contribute to improving the quality of the existing evidence.

 Research question: The authors have followed the PICO framework, but seem to have struggled to fit their research question into such a framework. Since this review does not consider a comparison, a PIO (population-intervention-outcome) or PEO (population-exposure-outcome) framework seems more appropriate. Furthermore, as no specific intervention is under study, the term “exposure” may be more fitting than “intervention.” I advise the authors to explore alternative frameworks for structuring their research question.

 Search strategy: I suggest including the Global Index Medicus database, which is free to consult and covers data from peripheral countries often underrepresented in mainstream databases. Also, authors should consider searching gray literature sources. I am aware that this decision might significantly increase the workload for the authors, but gray literature does provide valuable data for systematic reviews.

- Paez, A. (2017). Gray literature: An important resource in systematic reviews. Journal of Evidence‐Based Medicine, 10(3), 233-240.

Screening and selection: I see your literature search has retrieved over 8000 articles. This volume is exceedingly large for only two reviewers to manage, particularly during the full-text screening and data extraction phases. I recommend involving additional reviewers at later stages of the process, especially if the authors intend to publish the final review in 2025. Also, a third reviewer could be added for conflict resolution during the screening of abstracts, to reduce the workload during the full text stage.

 Data extraction: I recommend using a bigger sample for piloting of the data extraction form.

 Quality assessment: Does the MMAT checklist have cut-off points to cathegorize methodological quality? How are the authors planning to determine whether the quality of the studies is high or low? Moreover, the MMAT checklist is not comprehensive enough to determine risk of bias, which is necessary for a systematic review. Consider including other, more comprehensive tools, such as JBI’s (taking into account that the authors already use the JBI manual to guide their methodology).

 Data synthesis: Are the authors planning to conduct a meta-analysis of the quantitative data? If so, the methodology for this analysis should be clearly outlined.

 Public and patient involvement: Please clarify the role of the PPI panel in the review process. The current wording only states that they will be “consulted” during several stages, which is vague. A more explicit explanation of their specific contributions is needed.

Is the study design appropriate for the research question?

Yes

Is the rationale for, and objectives of, the study clearly described?

Yes

Are sufficient details of the methods provided to allow replication by others?

Partly

Are the datasets clearly presented in a useable and accessible format?

Not applicable

Reviewer Expertise:

Maternal health, breastfeeding determinants, epidemiology.

I confirm that I have read this submission and believe that I have an appropriate level of expertise to confirm that it is of an acceptable scientific standard, however I have significant reservations, as outlined above.

# Response

**Published:** 2025-11-19

**Authors:** Lucy Hackett

**Affiliations:** Trinity College Dublin School of Pharmacy and Pharmaceutical Sciences, Dublin, Leinster, Ireland

Many thanks for taking the time to review this protocol and for providing valuable feedback to improve the quality of our systematic review. We have revised our protocol following this and have individually addressed each comment below. Changes to the text are outlined in bold.

Reviewer comment 1:

 This mixed-methods systematic review protocol aims to explore the barriers and facilitators to self-management for women who consider breastfeeding during pharmacotherapy within the first two years postpartum. The rationale for this review is sufficiently detailed, as well as the gap in the evidence it aims to address.

**Response:**

 Thank you.

Reviewer comment 2:

 Background: When mentioning two previous reviews about the topic of breastfeeding and pharmacotherapy, I advise the authors to mention that none of these articles follow a systematic review methodology. Therefore, the proposed review would contribute to improving the quality of existing evidence.

**Response:**

 We have revised the final paragraph in the background to acknowledge this. Additionally, another systematic review on a similar topic has since been published (1), and thus has been discussed for further background and context to our review. The publication of this review has not indicated any changes to our methodological approach:

***‘Neither of these literature reviews have applied a systematic review methodology, which further emphasises the need for the proposed study. A recently published systematic review however (35), did explore one possible outcome for women who face concurrent breastfeeding and medication needs. This review aimed to identify the percentage of women who discontinue breastfeeding due to pharmacotherapy, which medications may trigger this, and other influencing factors. Authors of this review highlight the need for future research to explore women’s perspectives and experiences in these situations, their contact with healthcare professionals and services, and broader social or cultural contexts which may influence outcomes. Pilgrim and colleagues also suggest that future research use mixed methods designs and a theoretical framework to understand the determinants of women’s decision-making when faced with the need for medication while breastfeeding. The proposed review seeks to address this gap in the literature, and to address all possible outcomes and self-management decisions for women who face the simultaneous need for breastfeeding and medication, including avoidance or discontinuation of medication, or methods aiming to reduce infant exposure to medication through breastmilk.’***

Reviewer comment 3:

 Research question: The authors have followed the PICO framework, but seem to have struggled to fit their research question into such a framework. Since this review does not consider a comparison, a PIO (population-intervention-outcome) or PEO (population-exposure-outcome) framework seems more appropriate. Furthermore, as no specific intervention is under study, the term “exposure” may be more fitting than “intervention”. I advise the authors to explore alternative frameworks for structuring their research question.

**Response:**

 Thank you for this feedback. The use of the PICO question framework without identified comparators has been identified by both reviewers.  We have revised the paragraph regarding the research question framework in the methods section:

***‘The PEO (Population, Exposure, Outcome) tool is used to frame the research question*** ***(41)***
**
**

- ***Population: Studies which focus on the perspectives of mothers who breastfed their child(ren) at any point in the first two years of life, or women who considered breastfeeding. Studies which exclusively focus on the perspectives of healthcare professionals or other individuals will be excluded.***
- ***Exposure: Studies which address the need for simultaneous breastfeeding and pharmacotherapy. This may be understood as breastfeeding women who considered taking medication, or women who were undergoing pharmacotherapy and then considered breastfeeding. Medication in this context may be prescription-only medication, over-the-counter medication, or dietary supplements.***
- ***Outcome: The barriers and facilitators (or related concepts) to safe and effective self-management of medication use and breastfeeding, as experienced by mothers who face the need for these actions to be done concurrently.’***

Reviewer comment 4:

 Search strategy: I suggest including the Global Index Medicus database, which is free to consult and covers data from peripheral countries often underrepresented in mainstream databases. Also, authors should consider searching gray literature sources. I am aware that this decision might significantly increase the workload for the authors, but gray literature does provide valuable data for systematic reviews.

 Paez, A. (2017). Gray literature: An important resource in systematic reviews. Journal of Evidence‐Based Medicine, 10(3), 233-240.

**Response:**

 Thank you for the suggestions for how we can improve the search strategy of this review.

 A search was conducted using the Global Index Medicus database following this recommendation with guidance from a subject librarian to ensure this was done effectively. The following section in the search strategy was updated accordingly:

***‘Six electronic databases will be systematically searched: CINAHL (Cumulative Index to Nursing and Allied Health Literature), EMBASE (Excerpta Medica Database), PsycINFO, Medline (Ovid), Maternity and Infant Care, and Global Index Medicus.*** *Databases will be searched using terms related to medication, breastfeeding, women, experience, barriers, and facilitators. Searches will be limited to peer reviewed, empirical studies published in the English language, and not limited by year. Other literature reviews will be excluded. **The search strategies used for all six databases are available (39).**’*

 When designing the search strategy for this review, preliminary grey literature searches were carried out, with input from a subject librarian. Available grey literature sources returned hundreds of thousands of papers to be screened, which is unsurprising given the broad population and exposure. Considering the apparent abundance of published literature on the topic, it was decided to limit our literature search to peer-review published papers. Should our current search strategy reveal gaps in the published literature, this could then serve as a guide for a focussed search of grey literature to investigate these further.

 These decisions will be acknowledged as possible limitations in the final review if appropriate.

Reviewer comment 5:

 Screening and selection: I see your literature search has retrieved over 8000 articles. This volume is exceedingly large for only two reviewers to manage, particularly during the full-text screening and data extraction phases. I recommend involving additional reviewers at later stages of the process, especially if the authors intend to publish the final review in 2025. Also, a third reviewer could be added for conflict resolution during the screening of abstracts, to reduce the workload during the full text stage.

**Response:**

 Thank you for these suggestions. The following has been revised in the screening and selection section:

*‘Round 1 will comprise title and abstract screening. Dual independent screening will be carried out by two members of the research team to reduce bias and ensure high quality screening. **Disagreements will be resolved by consensus between the two reviewers or following input from a third reviewer if required.***

*Round 2 will comprise full text screening. Again, results will undergo dual independent screening by two researchers **. An additional reviewer may be added if necessary to facilitate screening in a timely manner. **Any discordance regarding the inclusion of a paper at this stage will be resolved by consensus between the two reviewers or following input from a third reviewer from the research team if required.’*

Reviewer comment 6:

 Data extraction: I recommend using a bigger sample for piloting of the data extraction form.

**Response:**

 Thank you for this feedback. This has been considered by the team and the data extraction form will now be piloted on five of the included studies. The following sentence in the protocol has been revised to reflect this:

*‘This form will be piloted on **five** studies identified from the list of included studies in the review and refined following this if necessary.’*

Reviewer comment 7:

 Quality assessment: Does the MMAT checklist have cut-off points to categorize methodological quality? How are the authors planning to determine whether the quality of the studies is high or low? Moreover, the MMAT checklist is not comprehensive enough to determine risk of bias, which is necessary for a systematic review. Consider including other, more comprehensive tools, such as JBI’s (taking into account that the authors already use the JBI manual to guide their methodology).

**Response:**

 Many thanks for your suggestions regarding this aspect of our review. Following your feedback, we (protocol authors) took time to re-examine both the MMAT and the JBI critical appraisal tools in detail, and this decision was the topic of several lengthy discussions within the research team. There is an acknowledgement in the literature that the terms ‘critical appraisal’, ‘quality assessment’ and ‘risk of bias’ are often used interchangeably (2, 3), with risk of bias used, mainly, in quantitative reviews (2). From an extensive review of the variety of tools/checklists used in other reviews, risk of bias appears be more applicable to quantitative, comparative reviews of effectiveness; given the purpose of our review, and the nature of the studies that we anticipate including, the risk of bias may not be as applicable. We considered the significant numbers of previously published systematic reviews which have used the MMAT effectively for critical appraisal (4-7). Alternative tools, such as the JBI critical appraisal tools, do not specifically account for mixed-methods research papers, which are likely to be included in our review. We considered the possibility of utilising both the JBI and MMAT tools – for example, using JBI critical appraisal tools for solely quantitative or qualitative papers, and using the MMAT for mixed-methods papers, as has been done in some published systematic reviews (8, 9). Additionally, we contemplated using the JBI critical appraisal tools alone, using a qualitative and quantitative assessment tool together to address the different aspects of any mixed-methods study included in our review. Considering the nature of our review, the need to ensure an appropriate tool for mixed-methods papers, and the desire to maintain consistency by appraising all papers with the same tool, the decision was reached that the best approach is to conduct critical appraisal using the MMAT.

 The MMAT advises against the calculation of an overall methodological quality score and removed this from their tool (10). Rather, they encourage users to provide more detail on the performance of the study against each methodological quality criterion to inform the quality of each study included in the review.

Reviewer comment 8:

 Data synthesis: Are the authors planning to conduct a meta-analysis of the quantitative data? If so, the methodology for this analysis should be clearly outlined.

**Response:**

 The authors do not plan to conduct a meta-analysis of the included quantitative data.   

Reviewer comment 9:

 Public and patient involvement: Please clarify the role of the PPI panel in the review process. The current wording only states that they will be “consulted” during several stages, which is vague. A more explicit explanation of their specific contributions is needed.

**Response:**

 The PPI panel were invited to validate the research question and the need for this systematic review prior to publication of this protocol. During the review process, PPI contributors will be involved in research tasks of their choice, with support and training, where needed. For example, they may be involved with interpreting study findings through participation in workshops, or in outreach activities to share the study findings.

**References:**

**1.** Pilgrim R, Kwok M, May A, Chapman S, Jones MD. The effect of medication use on breastfeeding continuation: a systematic review with narrative synthesis. *Int Breastfeed J*. 2025;20(1):59.

**2**. Stone JC, Barker TH, Aromataris E, Ritskes-Hoitinga M, Sears K, Klugar M, et al. From critical appraisal to risk of bias assessment: clarifying the terminology for study evaluation in JBI systematic reviews. *JBI Evid Synth.* 2023;21(3):472-7.

**3.** Furuya-Kanamori L, Xu C, Hasan SS, Doi SA. Quality versus Risk-of-Bias assessment in clinical research. *J Clin Epidemiol*. 2021;129:172-5.

**4.** Nawi AM, Ismail R, Ibrahim F, Hassan MR, Manaf MRA, Amit N, et al. Risk and protective factors of drug abuse among adolescents: a systematic review. *BMC Public Health. *2021;21(1):2088.

**5.** Bertolazzi A, Quaglia V, Bongelli R. Barriers and facilitators to health technology adoption by older adults with chronic diseases: an integrative systematic review. *BMC Public Health*. 2024;24(1):506.

**6.** Young AT, Amara D, Bhattacharya A, Wei ML. Patient and general public attitudes towards clinical artificial intelligence: a mixed methods systematic review. *Lancet Digit Health*. 2021;3(9):e599-e611.

**7.** Kucharczuk AJ, Oliver TL, Dowdell EB. Social media’s influence on adolescents′ food choices: A mixed studies systematic literature review. *Appetite.* 2022;168:105765.

**8.** Poon Y-SR, Lin YP, Griffiths P, Yong KK, Seah B, Liaw SY. A global overview of healthcare workers’ turnover intention amid COVID-19 pandemic: a systematic review with future directions. *Hum Resour Health*. 2022;20(1):70.

**9.** Mohammadifirouzeh M, Oh KM, Basnyat I, Gimm G. Factors associated with professional mental help-seeking among US immigrants: A systematic review. *J Immigr Minor Health.* 2023;25(5):1118-36.

**10.** Hong QN, Fàbregues S, Bartlett G, Boardman F, Cargo M, Dagenais P, et al.: The Mixed Methods Appraisal Tool (MMAT) version 2018 for information professionals and researchers. *Educ Inf.* 2018;34(4):285-91.
